# Short- and Long-Term Effects of Wholegrain Oat Intake on Weight Management and Glucolipid Metabolism in Overweight Type-2 Diabetics: A Randomized Control Trial

**DOI:** 10.3390/nu8090549

**Published:** 2016-09-07

**Authors:** Xue Li, Xiaxia Cai, Xiaotao Ma, Lulu Jing, Jiaojiao Gu, Lei Bao, Jun Li, Meihong Xu, Zhaofeng Zhang, Yong Li

**Affiliations:** 1 Department of Nutrition and Food Hygiene, School of Public Health, Peking University, Beijing 100191, China; xue.li@ed.ac.uk (X.L.); shuiruoran8886@126.com (X.C.); maxt2008@sina.com (X.M.); jingll_wit@bjmu.edu.cn (L.J.); jiaojiaogu442@gmail.com (J.G.); baolei6230@163.com (L.B.); xumeihong@bjmu.edu.cn (M.X.); zzfeng1104@bjmu.edu.cn (Z.Z.); 2 Centre for Population Health Sciences, University of Edinburgh, Edinburgh EH8 9AG, UK; 3 Department of Nutrition and Food Hygiene, School of Public Health, Capital Medical University, Beijing 100191, China; 4 Department of Clinical Nutrition, China-Japan Friendship Hospital, Peking University, Beijing 100191, China; 5 Department of Clinical Nutrition, International Hospital, Peking University, Beijing 100191, China; 6 The 153 Hospital of People’s Liberation Army, Zhengzhou 450001, China; pla153ywc@163.com

**Keywords:** type 2 diabetes, obesity, whole grain, oats, fiber

## Abstract

Glycemic control and weight reduction are primary goals for the management of overweight and obese type 2 diabetes mellitus (T2DM). Effective management cannot be achieved without an appropriate diet. Our study aimed to evaluate the short- and long-term effects of oat intake and develop a reasonable dietary plan for overweight T2DM patients. A randomized control trial, registered under ClinicalTrials.gov (Identification code: NCT01495052), was carried out among adult T2DM patients. A subgroup of 298 overweight subjects was selected and received a 30-day centralized intervention and 1-year free-living follow-up. Participants were randomly allocated to one of the following four groups. The usual care group (*n* = 60) received no intervention; the healthy diet group (*n* = 79) received a low-fat and high-fiber diet (“healthy diet”); the 50 g-oats group (*n* = 80) and 100 g-oats group (*n* = 79) received the “healthy diet” with the same amount of cereals replaced by 50 g and 100 g oats respectively. Anthropometric, blood glycemic and lipid variables were measured. For the 30-day intervention, significant differences in the changes of FPG (fasting plasma glucose), PPG (postprandial plasma glucose), HbA1c (glycosylated hemoglobin), HOMA-IR (homeostasis model assessment of insulin resistance), TC (total cholesterol), TG (total triglycerides), and LDL-c (low-density lipoprotein cholesterol) were observed among the four groups. Compared to the healthy diet group, the 50 g-oats group had a bigger reduction in PPG (mean difference (MD): −1.04 mmol/L; 95% CI: −2.03, −0.05) and TC (MD: −0.24 mmol/L; 95% CI: −0.47, −0.01); the 100 g-oats group had a bigger reduction in PPG (MD: −1.48 mmol/L; 95% CI: −2.57, −0.39), HOMA-IR (MD: −1.77 mU·mol/L^2^; 95% CI: −3.49, −0.05), TC (MD: −0.33 mmol/L; 95% CI: −0.56, −0.10) and LDL-c (MD: −0.22 mmol/L; 95% CI: −0.41, −0.03). In the 1-year follow-up, greater effects in reducing weight (MD: −0.89 kg; 95% CI: −1.56, −0.22), HbA1c (MD: −0.64%; 95% CI: −1.19, −0.09) and TG (MD: −0.70 mmol/L; 95% CI: −1.11, −0.29) were observed in the 100 g-oats group. In conclusion, short- and long-term oat intake had significant effects on controlling hyperglycemia, lowering blood lipid and reducing weight. Our study provided some supportive evidence for recommending oat as a good whole grain selection for overweight diabetics.

## 1. Introduction

Type 2 diabetes (T2DM) and obesity are both major global health problems, which have been linked with an increased risk of life-threatening comorbidities and enormous economic burdens [1,2]. Epidemiological studies report that most of the patients with T2DM are overweight or obese and, similarly, a significant number of obese individuals have diabetes [3]. This parallel prevalence indicates a strong association between T2DM and obesity. It has been further estimated that every 1 kilogram increase in body weight is associated with a 9% relative increase in diabetes prevalence [4]. Identification of this association has changed the primary goal of diabetes management in obese and overweight T2DM patients, and now controlling the blood glucose and reducing weight are both promoted [5]. 

Effective management of diabetes cannot be achieved without an appropriate diet, especially for type 2 diabetics who are overweight or obese. The recommended diet for controlling diabetes should be rich in dietary fiber, preferably provided by nature and less processed whole grains [6]. A Harvard study of health professionals found that high intake of whole grains was associated with lower incidence of T2DM [7] and therefore its benefit could be an important part of a diet which can help to improve diabetic control. 

Wholegrain foods can be found in a variety of cereals, but the content and solubility of fiber can vary significantly [8]. People with diabetes are often advised to select a good source of whole grains. Oat, with the advantage of having a high concentration of β-glucan, can be used for the management of diabetes [9]. The effects of oat intake have been investigated in several aspects [10,11,12]. Soluble fiber from oats has been found to be effective in lowering total cholesterol and low-density lipoprotein (5%–10% reduction with 3 g β-glucan intake per day), and thus oat and oat-products have already been recommended to patients with hyperlipidemia [13]. Besides, it has been suggested that oat intake can improve insulin response and decrease postprandial hyperglycemia [11,14]. Although these effects have been supported in many studies, others failed to replicate these. In particular, the effects of oat intake on fasting glucose concentration and weight control remain conflicting [11,14]. Therefore, further work is needed to determine whether oat intake has the reported benefits and whether these benefits could be observed similarly in specific populations, particularly in overweight T2DM patients. 

To develop a reasonable dietary plan, which includes wholegrain oats, a randomized control trail was conducted among adults with T2DM in Baotou, Inner Mongolia, China. This study aimed to compare the short- and long-term integrative effects of oat intake with a low-fat and high-fiber diet (“healthy diet”) on weight management, blood glucose control and lipid-profile improvement in overweight T2DM patients. 

## 2. Methods

### 2.1. Participants

**Figure 1:**
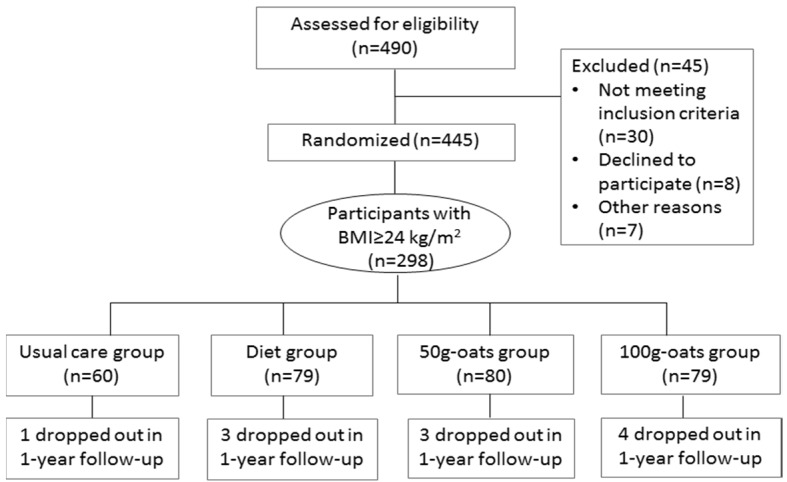
Flow chart for subject enrollment, allocation, intervention and follow-up.

A subgroup of 298 subjects, meeting the Chinese criteria of overweight (body mass index ≥ 24 kg/m^2^), was selected from 445 adult patients with T2DM, who had participated in the 30-day centralized management of a dietary program and the 1-year free-living follow-up in Baotou, China. The sample size of the original study was calculated based on an estimated standard deviation (SD) of 2.7 mmol/L in HbA1c. A total of 420 participants were required to detect a difference of 0.80 SD of HbA1c with 90% power and allowing for 10% missing data. Individuals who were heavy smokers (smoking more than or equal to 25 cigarettes per day) or heavy drinkers (drinking more than 25 mL alcohol per day), or had recent changes (less than 3 months) in diet and physical activities, or had severe cardiovascular, renal or hepatic complications, mental illness or other serious diseases, or recently accepted glucocorticoid treatment, or had already been eating oats or oat products as part of their diet, were excluded. At the end of recruitment, a total of 445 individuals were included and randomized, of which 298 overweight participants were selected for this subgroup analysis. Eleven patients dropped out during the 1-year follow-up due to personal reasons with no difference in drop-out rates among the four groups (*p* = 0.774) (Figure 1).

### 2.2. Ethics

This study was approved by the ethic review board of China-Japan Friendship Hospital of Health Ministry China in December 2011 and registered under ClinicalTrials.gov (Identification code: NCT01495052 available at https://clinicaltrials.gov/). Written and oral information of the study protocol was given prior to the study initiation. Informed consent was signed by every participant. 

### 2.3. Study Design

During the 30-day centralized intervention, all participants were arranged to live in a hotel and have meals together under the supervision of 12 qualified dietitians and 18 well trained investigators. Food intake and compliance of participants were recorded every day by the investigators. Physical activities were assessed and categorized using the recommendations of the international physical activity questionnaire (IPAQ). Participants were required to record uncomfortable symptoms and maintain their normal physical activities and medications. After a one-week run-in period, participants were randomly allocated to one of the following four groups by computer-generated random numbers. The usual care group (*n* = 60) served as the control group and received no dietry intervention. They took meals depending on their own eating habits. The healthy diet group (*n* = 79) received a low-fat and high-fiber diet (“healthy diet”). A 7-day cyclical menu (Supplementary Materials Table S1) was designed according to the China Food Composition [15], the Dietary Guidelines for Chinese Resident [16] and the China Medical Nutrition Therapy Guideline for Diabetes [17] to provide a low-fat and high-fiber diet. Each participant was provided with three meals a day, which contained 2275 kcal for men and 1890 kcal for women (60% from carbohydrate, 22% from fat, 18% from protein) and 30 g of dietary fiber. In addition, a maximum of an extra 10% of daily kcal intake was allowed according to the individual need of participants. For the 50 g and 100 g-oats groups, participants received the “healthy diet” with the same amount of cereals replaced by 50 g and 100 g of wholegrain oats respectively. The daily intake of energy and macronutrients for each group is shown in Table 1. Apart from the dietary intervention, the study dieticians gave nutritional education and training to the three intervention groups six times per week to encourage the participants to have a general healthy diet in daily life.

After the centralized management, all participants returned home and were asked to continue with their intervention and record their daily diet, uncomfortable symptoms and medication changes. Wholegrain oats were continuously provided for the 50 g and 100 g-oats groups. Investigators continued to give diet recommendations and supervise the daily life of participants by monthly group interviews through network-chat, telephone or face-to-face interviews. Scheduled clinical checks were performed every three months. The follow-up lasted for one year.

### 2.4. Wholegrain Oats

The wholegrain oats used in this study were provided by Inner Mongolia Sanzhuliang Natural Oats Industry Corporation (IMSNOIC) (Hohhot, Inner Mongolia, China). The oats were grown in northwest China and were processed by a peeling technology which retained the necessary ingredients and beneficial nutrients of the whole grain [18]. The nutrient composition of this product was analyzed by the laboratory of the School of Life Sciences, Sun Yat-sen University, according to a standard procedure (GB/T 5009). Each 100 g of wholegrain oats contained 63.5 g carbohydrate, 7.6 g fat, 13.7 g protein, and 8.7 g fiber, of which approximately 5.3 g was β-glucan.

### 2.5. Outcome Measurement

Physical examinations were performed at baseline, at the end of the 30-day intervention and at the end of the 1-year follow-up. Anthropometric measurements were carried out for weight, height, waist and hip circumference, and blood pressure. Body fat percent and visceral fat index (VFI) were measured using bioelectrical impedance scales (Tanita BF-622W, Tanita Corporation of the United State). Venus blood samples were collected after an overnight fast for testing fasting plasma glucose (FPG), 2-h postprandial plasma glucose (PPG), glycosylated hemoglobin (HbA1c), fasting plasma insulin, 2-h postprandial plasma insulin, total triglycerides (TG), total cholesterol (TC), low-density lipoprotein cholesterol (LDL-c), and high-density lipoprotein cholesterol (HDL-c). Insulin resistance was calculated by the formula: HOMA-IR = fasting serum insulin (μU/mL) × FPG (mmol/L)/22.5. All measurements were conducted with standard procedures by the same clinical staff in the third hospital of Inner Mongolia medical college, who were blinded to the group allocation.

### 2.6. Statistical Analyses

Categorical and continuous variables were analyzed by either Chi-squared test or sample *t*-test. Responses to interventions were assessed by the changes in anthropometric and metabolic variables, determined at baseline and at the end of the intervention. A generalized linear model (GLM) was applied to estimate the changes after adjusting for potential confounding factors including sex, age, drinking, smoking, physical activity level, education level, family history of diabetes, diabetic medications and the duration of diabetes. The mean differences (MD) of changes among groups were calculated to compare the effects of different interventions. We performed multiple imputations to account for missing data (SAS Institute, Inc., Cary, NC, USA). Results are presented as means with standard deviation (SD) or 95% confidence intervals (95% CI). All tests were two-sided and *p* < 0.05 was considered to be statistically significant. Analyses were conducted with the IBM SPSS Statistics 22 (IBM Corp., Armonk, NY, USA, 2013), unless otherwise stated.

## 3. Results

The main characteristics of the participants in each group are shown in Table 2. Among the four groups, there were no significant differences in the baseline characteristics of sex, age, drinking, smoking, physical activity, education level, the duration of diabetes, family history of diabetes, diabetic medications and blood pressure. 

Changes in variables, and the mean differences of changes among the four groups after the 30-day intervention, are presented in Table 3. When compared to the baseline values of the anthropometric variables, the three intervention groups had a significant reduction in weight, BMI and waist circumference, while significant reduction in visceral fat index (VFI) was only observed in the 50 g oats group (adjusted change: −0.48; 95% CI: −0.69, −0.27) and the 100 g oats group (adjusted change: −0.44; 95% CI: −0.78, −0.10). When comparing the mean differences between groups, there were no statistically significant differences in the changes of anthropometric variables between the healthy diet group and the oats groups. 

For the glycemic variables, a significant reduction in FPG, PPG and HbA1c from baseline was observed in the three intervention groups, while a significant decrease in HOMA-IR was observed in 50 g oats group (adjusted change: −1.80 mU·mol/L^2^; 95% CI: −3.48, −0.12) and 100 g oats group (adjusted change: −2.65 mU·mol/L^2^; 95% CI: −4.72, −0.58). When comparing the changes between groups (the healthy diet group was the reference), the 50 g oats group showed a bigger reduction in PPG (MD: −1.04 mmol/L; 95% CI: −2.03, −0.05), and the 100 g oats group showed a bigger reduction in PPG (MD: −1.48 mmol/L; 95% CI: −2.57, −0.39) and HOMA-IR (MD: −1.77 mU·mol/L^2^; 95% CI: −3.49, −0.05). 

Compared to the baseline values of the lipid variables, the three intervention groups had a significant reduction in TC and LDL-c. When comparing between groups, the 50 g oats group had a bigger reduction in TC (MD: −0.24 mmol/L; 95% CI: −0.47, −0.01) and the 100 g oats group had a bigger reduction in TC (MD: −0.33 mmol/L; 95% CI: −0.56, −0.10) and LDL-c (MD: −0.22 mmol/L; 95% CI: −0.41, −0.03) than the healthy diet group. For TG, no statistically significant difference in the reduction was observed in the two oats groups when compared to the healthy diet group. 

The changes and mean differences of changes in variables between the baseline and the end of the 1-year intervention are shown in Table 4. Compared with the baseline values, the three intervention groups had significant reduction in FPG, PPG, HbA1c, TC, and LDL-c, while the significant decrease in weight, BMI and TG was only observed in the 50 g and 100 g oats groups. Comparing the oats groups to the healthy diet group, the 50 g oats group had a bigger reduction in TG (MD: −0.42 mmol/L; 95% CI: −0.83, −0.01) and LDL-c (MD: −0.27 mmol/L; 95% CI: −0.49, −0.05), and the 100 g oats group had a bigger reduction in weight (MD: −0.89 kg; 95% CI: −1.56, −0.22), PPG (MD: −1.17 mmol/L; 95% CI: −2.27, −0.07), HbA1c (MD: −0.64%; 95% CI: −1.19, −0.09), TC (MD: −0.30 mmol/L; 95% CI: −0.57, −0.03), TG (MD: −0.70 mmol/L; 95% CI: −1.11, −0.29), and LDL-c (MD: −0.37 mmol/L; 95% CI: −0.59, −0.15). 

## 4. Discussion

The present study showed that a low-fat and high-fiber diet (“healthy diet”) had beneficial effects on glucolipid metabolism in overweight T2DM patients, and these effects were more evident when combined with oat intake. In particular, the combination of short-term (30 days) oat intake with the “healthy diet” had greater effects on lowering the PPG, HOMA-IR, TC and LDL-c than that of merely having a low-fat and high-fiber diet. The 1-year follow-up showed that the reduction of PPG, HOMA-IR, TC and LDL-c can be maintained for long time, and significantly greater effects in decreasing weight, HbA1c and TG were observed. 

The primary finding of this study was the significant effect of oat intake on hyperglycemia control. A growing number of studies have suggested that oats and oat-enriched products can significantly decrease the postprandial hyperglycemia [11,14]. Consistently, our study provided supportive evidence for the PPG lowering effect of oat intake in overweight T2DM patients. Although the mechanism of lowering PPG has not been fully understood, at least parts of the contribution could be attributed to the property of oat β-glucan. Oat β-glucan can increase the viscosity in the intestine, slow the absorption of carbohydrates, and thus reduce the PPG [19,20]. Research findings on the FPG lowering effects of oat intake are less consistent. A few human trials and diabetic mice laboratory studies have found that oat intake can significantly decrease the FPG concertation [14,21,22], but this finding was not supported by the majority of randomized control trials (RCTs) [11]. Although a subgroup analysis of high quality RCTs in a meta-analysis indicated that oat intake can slightly lower FPG concentration in the long-term intervention [11], our study did not find any FPG lowering effect that could be attributed to either short-term or long-term oat intake. The long-term oat intake had a significant effect in reducing HbA1c, but short-term oat intake did not. This finding is not totally unexpected. Considering that HbA1c levels usually reflect the blood glucose levels for the period of 8–12 weeks, the duration of a 30-day intervention may not be long enough to have significant changes in HbA1c, whereas a significant reduction in HbA1c could be shown in the longer 1-year follow-up. So far, a few studies have evaluated the effect of oat intake on HOMA-IR. A meta-analysis of three RCTs reported that oat intake had no effect on the improvement of HOMA-IR [11]. In contrast, our study suggested a significant effect on decreasing HOMA-IR. Our finding was consistent with a recent published RCT, which suggested oats consumption can significantly decrease the HOMA-IR index [23]. These varying results may be partly due to the different characteristics of study populations, probably because overweight and obese T2DM patients are likely to result in more severe insulin resistance, and insulin resistance is more likely to be modifiable in this population [24]. 

Furthermore, this study confirmed the serum lipid lowering effect of oat intake in overweight T2DM patients. The effect of oat intake on lowering TC and LDL-c has been supported in most published studies [10,13]. The main controversy is the magnitude of the effect. In some studies, the reduction of TC and LDL-c could be more than 10% [25,26], while in others, the reduction was less than 5% [27,28]. In our study, the decrease of TC and LDL-c was around 10%. However, the effect size in this study was not comparable with others, considering the study population, the dose of oats and oat processing were different from other studies. Comparing the duration of the intervention, the long-term oat intake had a significant effect on the reduction of TG. The mechanism of serum lipid-lowering effect also seems to be related to the increased viscosity attributed to the oat β-glucan, which can lead to the reduction in cholesterol absorption [20]. 

Another finding which is important for overweight T2DM patients is the weight loss effect of long-term oat intake. During the 30-day intervention, the three intervention groups had a significant decrease in body weight and the weight reduction was similar among the three diet groups. However, during the 1-year follow-up, the 100 g oats group had a significantly greater decrease in weight than the healthy diet group. Considering that oat intake was combined with the “healthy diet”, it is possible that the moderate weight-reducing effect of a short-term (30 days) oat intake was covered up by the weight reduction due to the low-fat and high-fiber diet; or the duration of a 30-day oat intake was too short to have changes on weight. The bigger weight reduction observed in the 1-year oat intake was unlikely to be caused by the poorly controlled blood glucose level, since a significant reduction in FPG, PPG and HbA1c was also observed. The weight reduction effect is probably related to the oat β-glucan, which may enhance the viscosity of meals, decrease the starch digestion and reduce the food intake by increasing satiety [29]. In addition to our studies, a few other studies also indicated a weigh-reducing effect of oats in certain populations [23,29,30], but these findings were not consistently in agreement, as some studies found no decrease in weight [12,25,31]. Further research is needed to verify the weight-reducing effect of oat intake.

The predominant effect of oats on diabetic management is most likely to be attributed to the bioactivity of β-glucan. Although, compared to other cereals, wholegrain oats have distinct bioactive composition in lipids and phenolics, the most significant difference is in the high content of β-glucan, especially when considering that the effective dose of the other two components is not likely to be achieved through 50 g or 100 g wholegrain oats consumption [30,31]. As mentioned above, β-glucan has been reported to increase the intestinal viscosity, decrease the absorption of carbohydrates and lipids, and reduce food intake to control hyperglycemia, lower lipid and reduce weight. In addition, another important role of β-glucan involves the impact on gut microbiota. Specifically, the bacterial metabolism of β-glucan can increase the production of short-chain fatty acids and drive the release of bioactive compounds, which may interact with host biology to affect the risk of obesity and associated disorders [32,33]. Oat β-glucan has been shown to decrease the protein fermentation and thus reduce the detrimental metabolites produced [34]. Furthermore, the fermentation of β-glucan has also been reported to increase the diversity of gut microbiota, which is a potential benefit, considering that the reduced microbiota diversity is associated with obesity [35,36]. Studies exploring the mechanisms behind the health benefits of β-glucan and gut microbiota may provide more evidence to encourage an increase in oat intake and to maximize the health benefits derived from oats. 

Strengths and limitations of the study design should be noted. In the first phase, a 30-day centralized management of intervention was designed to improve the compliance of participants. Potential dietary confounding factors for assessing the effects of oat intake were homogenized among groups by providing a general “healthy diet” to the intervention groups of oat intake and using a healthy diet group as the reference group. To estimate the beneficial effects due to the “healthy diet”, a usual care group, with no dietry intervention, was established as a control group. To provide information for recommending a proper dose of daily oat intake (or oat β-glucan), we designed two intervention groups with different doses (50 g and 100 g) of oat intake. Considering that the duration of a 30-day intervention is probably too short for some variables, such as HbAc1 and weight, to have significant changes, a 1-year follow-up was designed to investigate the long-term changes of the variables and to determine if the significant changes observed in the 30-day intervention could be maintained for a long time. However, in this subgroup analysis, we only reported that the anthropometric and blood biochemical variables, cardiovascular events and other diabetic complications were not presented, which led us to consider that the duration of a 1-year intervention was relatively short for evaluating the diabetic complications. Another limitation was that the subjects were not fully blinded because of the different taste of oats and other cereals. Furthermore, the benefits of oat intake were assessed in comparison to an already healthy diet, and as discussed above, it is possible that the moderate beneficial effects of oat intake were covered up or magnified by the healthy reference diet. However, in either case, it should be emphasized that the whole grain oat intake should be recommended with a healthy diet. 

## 5. Conclusions

In conclusion, our study provided some supportive evidence that oats can be a good selection of whole grains for overweight diabetics, but further larger scale studies are needed to evaluate these findings further.

## Figures and Tables

**Table 1:** The daily intake of energy and macronutrients (one day’s intake).

Dietary Components	Usual Care Group	Treatment Diet		
		Healthy Diet Group	50 g-oats Group	100 g-oats Group
Energy (kcal) *	2441 (478)	2279 (196)	2281 (185)	2233 (204)
Carbohydrate (% of total energy)	50	60	59	58
Fat (% of total energy)	31	22	23	23
Protein (% of total energy)	19	18	18	19
Total fiber (g) *	22.1 (4.0)	33.0 (5.8)	36.1 (4.2)	39.0 (4.8)
Oat β-glucan (g)	0	0	2.65	5.30

* Variables are presented as mean with (SD (standard deviation)).

**Table 2:** The baseline characteristics of the study participants *.

Variables	Usual Care Group (*n* = 60)	Healthy Diet Group (*n* = 79)	50 g-oats Group (*n* = 80)	100 g-oats Group (*n* = 79)	*p*-Value
Male/female	39/21	42/37	41/39	33/46	0.059
Age (years)	59.00 (3.94)	59.73 (6.53)	59.72 (6.10)	59.44 (6.78)	0.886
Mild drinking	15 (25.0%)	20 (25.3%)	17 (21.3%)	16 (20.3%)	0.838
Mild smoking	14 (23.3%)	16 (20.3%)	14 (17.5%)	11 (13.9%)	0.523
Physical activity level	-	-	-	-	0.541
Low	13 (21.7%)	18 (22.8%)	17 (21.2%)	22 (27.8%)	-
Moderate	30 (50.0%)	42 (53.1%)	45 (56.3%)	46 (58.2%)	-
High	17 (28.3%)	19 (24.1%)	18 (22.5%)	11 (13.9%)	-
Education level	-	-	-	-	0.473
Less than primary school	9 (15.0%)	9 (11.4%)	15 (18.8%)	8 (10.1%)	-
Middle and high school	39 (65.0%)	56 (70.9%)	47 (58.8%)	49 (62.0%)	-
College or more	12 (20.0%)	14 (17.7%)	18 (22.5%)	22 (27.8%)	-
Duration of diabetes (month)	79.00 (36.52)	74.87 (61.92)	100.08 (75.73)	94.71 (76.63)	0.060
Family history of diabetes	19 (31.7%)	24 (30.4%)	36 (45.0%)	38 (48.1%)	0.051
Diabetic medications	-	-	-	-	0.999
No diabetic medication	5 (8.3%)	7 (8.9%)	6 (7.5%)	6 (7.6%)	-
Oral diabetic medication	32 (53.3%)	45 (57.0%)	43 (53.8%)	47 (59.5%)	-
Insulin injection	12 (20.0%)	14 (17.7%)	16 (20.0%)	12 (15.2%)	-
Combined treatment	11 (18.3%)	13 (16.4%)	15 (18.7%)	14(17.7%)	-
Systolic blood pressure (mmHg)	143.71 (15.83)	147.23 (21.31)	144.90 (19.18)	147.19 (17.68)	0.613
Diastolic blood pressure (mmHg)	84.43 (16.05)	84.63 (11.78)	82.93 (9.39)	83.10 (10.20)	0.737

* Continuous variables are presented as mean with (SD) and categorical variables are presented as a number (with percentage).

**Table 3:** Changes in variables and mean differences (MD) in changes among groups after 30-day intervention *.

Variables	Usual Care Group (*n* = 60)	Healthy Diet Group (*n* = 79)	50 g-oats Group (*n* = 80)	100 g-oats Group (*n* = 79)	*p*-Value
Weight (kg)					
Baseline	71.54 (5.82)	73.77 (8.58)	72.60 (8.67)	74.44 (7.63)	0.141
30-day intervention	71.45 (6.00)	72.59 (7.94)	71.74 (8.50)	72.70 (7.21)	-
Adjusted changes	−0.18 (−1.39, 1.02)	−1.20 (−2.22, −0.19)	−1.67(−2.69, −0.65)	−1.74 (−2.76, −0.71)	0.178
MD (vs. usual care group)	-	−1.02 (−2.56, 0.52)	−1.49 (−3.03, 0.05)	−1.56 (−3.14, 0.02)	-
MD (vs. diet group)	-	-	−0.47 (−1.89, 0.96)	−0.54 (−1.97, 0.89)	-
BMI (kg/m^2^)					
Baseline	25.17 (0.89)	27.19 (2.82)	26.91 (2.69)	27.39 (2.42)	0.000
30-day intervention	25.14 (0.94)	26.77 (2.66)	26.28 (3.86)	26.77 (2.33)	-
Adjusted changes	−0.08 (−0.49, 0.33)	−0.43 (−0.78, −0.08)	−0.60(−0.95, −0.25)	−0.63 (−0.98, −0.28)	0.160
MD (vs. usual care group)	-	−0.35 (−0.88, 0.18)	−0.52 (−1.05, 0.01)	−0.55 (−1.10, 0.00)	-
MD (vs. diet group)	-	-	−0.17 (−0.66, 0.32)	−0.20 (−0.69, 0.29)	-
Waist circumference (cm)					
Baseline	92.69 (7.94)	94.81 (7.01)	93.38 (6.79)	94.86 (7.65)	0.210
30-day intervention	91.92 (8.22)	92.02 (8.63)	91.08 (3.98)	92.07 (7.27)	-
Adjusted changes	−0.78 (−2.92, 1.36)	−2.79 (−3.85, −1.73)	−2.32 (−3.37, −1.27)	−2.77 (−3.83, −1.70)	0.028
MD (vs. usual care group)	-	−2.01 (−3.54, −0.48)	−1.54 (−3.08, 0.00)	−1.99 (−3.48, −0.50)	-
MD (vs. diet group)	-	-	0.47 (−1.01, 1.95)	0.02 (−1.47, 1.50)	-
Waist-to-hip ratio(WHR)					
Baseline	0.91 (0.05)	0.92 (0.05)	0.91 (0.05)	0.92 (0.05)	0.398
30-day intervention	0.90 (0.05)	0.91(0.07)	0.90 (0.05)	0.90 (0.05)	-
Adjusted changes	−0.01(−0.02, 0.01)	−0.01 (−0.02, 0.01)	−0.01 (−0.02, 0.01)	−0.02 (−0.03, −0.01)	0.259
MD (vs. usual care group)	-	0.00 (−0.02, 0.02)	0.00 (−0.02, 0.02)	−0.01 (−0.03, 0.01)	-
MD (vs. diet group)	-	-	0.00 (−0.02, 0.02)	0.00 (−0.02, 0.02)	-
Body fat percent (%)					
Baseline	32.46 (5.49)	31.58 (6.11)	31.54 (5.87)	33.31 (5.12)	0.162
30-day intervention	32.21 (5.88)	31.05 (6.23)	31.37 (5.75)	32.78 (5.34)	-
Adjusted changes	−0.25 (−1.09, 0.59)	−0.59 (−1.20, 0.02)	−0.52 (−1.12, 0.08)	−0.52 (−1.13, 0.08)	0.878
MD (vs. usual care group)	-	−0.34 (−1.06, 0.36)	−0.27 (−0.85, 0.31)	−0.27 (−0.81, 0.26)	-
MD (vs. diet group)	-	-	0.07 (−0.51, 0.65)	0.07 (−0.51, 0.65)	-
Visceral fat index (VFI)					
Baseline	12.37 (3.64)	12.38 (3.86)	12.53 (4.14)	12.33 (3.59)	0.988
30-day intervention	12.13 (3.77)	12.13 (3.53)	12.06 (4.42)	11.87 (3.47)	-
Adjusted changes	−0.25 (−0.55, 0.05)	−0.27 (−0.62, 0.07)	−0.48 (−0.69, −0.27)	−0.44 (−0.78, −0.10)	0.380
MD (vs. usual care group)	-	−0.02 (−0.44, 0.40)	−0.24 (−0.74, 0.26)	−0.19 (−0.73, 0.35)	-
MD (vs. diet group)	-	-	−0.22 (−0.70, 0.27)	−0.17 (−0.64, 0.31)	-
Fasting plasma glucose (mmol/L)					
Baseline	9.38 (2.81)	9.52 (2.87)	9.87 (2.83)	9.70 (3.30)	0.719
30-day intervention	9.40 (0.75)	8.16 (2.53)	8.67 (2.49)	8.03 (2.56)	-
Adjusted changes	−0.20 (−0.91, 0.52)	−1.27 (−1.88, −0.67)	−1.23 (−1.84, −0.62)	−1.70 (−2.31, −1.10)	0.002
MD (vs. usual care group)	-	−1.07 (−1.99, −0.15)	−1.03 (−1.94, −0.11)	−1.50 (−2.42, −0.58)	-
MD (vs. diet group)	-	-	0.04 (−0.81, 0.89)	−0.43 (−1.28, 0.42)	-
2-h postprandial plasma glucose (mmol/L)					
Baseline	19.10 (3.22)	17.58 (4.87)	18.23 (4.84)	17.89 (5.45)	0.284
30-day intervention	18.66 (3.07)	15.42 (4.31)	14.97 (4.10)	14.08 (4.62)	-
Adjusted changes	−0.53 (−1.45, 0.39)	−2.14 (−2.92, −1.36)	−3.18 (−3.95, −2.41)	−3.62 (−4.39., −2.84)	0.001
MD (vs. usual care group)	-	−1.61 (−2.79, −0.43)	−2.65 (−3.82, −1.47)	−3.09 (−4.27, −1.91)	-
MD (vs. diet group)	-	-	−1.04 (−2.03, −0.05)	−1.48 (−2.57, −0.39)	-
HbA1c (%)					
Baseline	8.05 (1.52)	8.10 (1.77)	8.37 (1.44)	8.28 (1.35)	0.463
30-day intervention	8.07 (1.52)	7.88 (1.82)	7.71 (1.94)	7.65 (1.93)	-
Adjusted changes	0.10 (−0.34, 0.54)	−0.61 (−0.98, −0.24)	−0.76 (−1.13, −0.39)	−0.71 (−1.09, −0.34)	0.001
MD (vs. usual care group)	-	−0.71 (−1.29, −0.13)	−0.86 (−1.43, −0.29)	−0.81 (−1.37, −0.24)	-
MD (vs. diet group)	-	-	−0.14 (−0.67, 0.39)	−0.10 (−0.63, 0.43)	-
HOMA-IR (mU·mol/L^2^)					
Baseline	5.49 (4.99)	5.48 (5.40)	4.68 (3.78)	6.20 (5.78)	0.312
30-day intervention	5.31 (3.16)	4.50 (4.89)	3.41 (3.23)	3.76 (4.75)	-
Adjusted changes	−0.25 (−2.66, 2.16)	−0.89 (−3.52, 1.74)	−1.80 (−3.48, −0.12)	−2.65 (−4.72, −0.58)	0.010
MD (vs. usual care group)	-	−0.64 (−2.40, 1.12)	−1.55 (−3.30, 0.20)	−2.41 (−4.59, −0.23)	-
MD (vs. diet group)	-	-	−0.91 (−1.93, 0.11)	−1.77 (−3.49, −0.05)	-
TC (total cholesterol) (mmol/L)					
Baseline	5.84 (1.83)	4.98 (0.86)	5.04 (0.98)	5.24 (1.03)	0.000
30-day intervention	5.82 (1.88)	4.81 (0.87)	4.66 (0.87)	4.72 (0.85)	-
Adjusted changes	−0.07 (−0.26, 0.12)	−0.18 (−0.34, −0.02)	−0.42 (−0.59, −0.26)	−0.51 (−0.67, −0.35)	0.000
MD (vs. usual care group)	-	−0.11 (−0.35, 0.14)	−0.35 (−0.60, −0.10)	−0.44 (−0.69, −0.19)	
MD (vs. diet group)	-	-	−0.24 (−0.47, −0.01)	−0.33 (−0.56, −0.10)	-
TG (total triglycerides) (mmol/L)					
Baseline	1.92 (0.94)	1.83 (0.88)	2.06 (1.06)	1.98 (1.00)	0.510
30-day intervention	1.95 (1.02)	1.57 (0.84)	1.98 (1.65)	1.56 (0.77)	-
Adjusted changes	0.01 (−0.25, 0.27)	−0.25 (−0.47, −0.03)	−0.09 (−0.31, 0.13)	−0.43 (−0.65, −0.21)	0.003
MD (vs. usual care group)	-	−0.26 (−0.60, 0.08)	−0.10 (−0.43, 0.23)	−0.44 (−0.78, −0.10)	-
MD (vs. diet group)	-	-	0.16 (−0.26, 0.58)	−0.17 (−0.59, 0.26)	-
LDL-c (low-density lipoprotein cholesterol) (mmol/L)					
Baseline	3.20 (1.05)	2.96 (0.71)	2.90 (0.77)	3.15 (0.85)	0.128
30-day intervention	3.18 (1.05)	2.85 (0.74)	2.70 (0.70)	2.79 (0.63)	-
Adjusted changes	−0.06 (−0.21, 0.10)	−0.12 (−0.25, 0.01)	−0.23 (−0.36, −0.10)	−0.34 (−0.47, −0.21)	0.001
MD (vs. usual care group)	-	−0.06 (−0.26, 0.14)	−0.17 (−0.37, 0.02)	−0.28 (−0.48, −0.08)	-
MD (vs. diet group)	-	-	−0.10 (−0.28, 0.08)	−0.22 (−0.41, −0.03)	-
HDL-c (high-density lipoprotein cholesterol) (mmol/L)					
Baseline	1.41 (0.45)	1.30 (0.24)	1.25 (0.21)	1.36 (0.36)	0.022
30-day intervention	1.39 (0.42)	1.22 (0.24)	1.20 (0.22)	1.28 (0.26)	-
Adjusted changes	−0.02 (−0.07, 0.04)	−0.08 (−0.19, 0.03)	−0.07 (−0.11, −0.02)	−0.08 (−0.13, −0.03)	0.635
MD (vs. usual care group)	-	−0.06 (−0.14, 0.01)	−0.05 (−0.12, 0.02)	−0.06 (−0.14, 0.02)	-
MD (vs. diet group)	-	-	0.01 (−0.05, 0.07)	0.00 (−0.07, 0.07)	-

* All values were presented as means (SD) or means (95% CI). Changes from the baseline were adjusted for potential confounding variables (sex, age, drinking, smoking, physical activity level, education level, family history of diabetes, diabetic medications and duration of diabetes) in the analysis of covariance model.

**Table 4:** Changes in variables and mean differences (MD) in changes among groups after 1-year follow-up *.

Variables	Usual Care Group (*n* = 59)	Healthy Diet Group (*n* = 76)	50 g-oats Group (*n* = 77)	100 g-oats Group (*n* = 75)	*p*-Value
Weight (kg)					
Baseline	71.54 (5.82)	73.77 (8.58)	72.60 (8.67)	74.44 (7.63)	0.141
1-year follow-up	71.47 (7.35)	72.76 (8.70)	71.39 (8.68)	72.43 (7.58)	-
Adjusted changes	−0.11 (−0.68, 0.46)	−1.08 (−2.31, 0.16)	−1.44 (−2.74, −0.15)	−1.97 (−3.06, −0.88)	0.012
MD (vs. usual care group)	-	−0.97 (−2.23, 0.29)	−1.33 (−2.69, 0.03)	−1.86 (−3.25, −0.47)	-
MD (vs. diet group)	-	-	−0.36 (−1.10, 0.38)	−0.89 (−1.56, −0.22)	-
BMI (kg/m^2^)					
Baseline	25.17 (0.89)	27.19 (2.82)	26.91 (2.69)	27.39 (2.42)	0.000
1-year follow-up	25.13 (1.25)	26.80 (2.91)	26.48 (2.33)	26.64 (2.74)	-
Adjusted changes	−0.05 (−0.39, 0.29)	−0.37 (−0.74, 0.00)	−0.50 (−0.91, −0.09)	−0.73 (−1.08, −0.39)	0.128
MD (vs. usual care group)	-	−0.33 (−1.05, 0.39)	−0.45 (−1.19, 0.29)	−0.68 (−1.43, 0.07)	-
MD (vs. diet group)	-	-	−0.12 (−0.64, 0.40)	−0.33 (−0.83, 0.17)	-
Fasting plasma glucose (mmol/L)					
Baseline	9.38 (2.81)	9.52 (2.87)	9.87 (2.83)	9.70 (3.30)	0.719
1-year follow-up	9.52 (1.44)	7.94 (2.14)	8.19 (2.01)	7.74 (2.43)	-
Adjusted changes	0.08 (−0.63, 0.46)	−1.65 (−2.21, −1.10)	−1.62 (−2.17, −1.07)	−1.87 (−2.44, −1.31)	0.000
MD (vs. usual care group)	-	−1.57 (−2.36, −0.78)	−1.54 (−2.33, −0.75)	−1.79 (−2.58, −1.00)	-
MD (vs. diet group)	-	-	0.03 (−0.76, 0.82)	−0.22 (−1.02, 0.58)	-
2-h postprandial plasma glucose (mmol/L)					
Baseline	19.10 (3.22)	17.58 (4.87)	18.23 (4.84)	17.89 (5.45)	0.284
1-year follow-up	19.69 (3.27)	15.01 (3.63)	14.98 (3.02)	14.22 (3.78)	-
Adjusted changes	0.63 (−0.36, 1.63)	−2.41 (−3.40, −1.42)	−3.16 (−4.16, −2.16)	−3.58 (−4.63, −2.53)	0.000
MD (vs. usual care group)	-	−3.04 (−4.48, −1.60)	−3.79 (−5.22, −2.36)	−4.21 (−5.67, −2.75)	-
MD (vs. diet group)	-	-	−0.75 (−1.91, 0.41)	−1.17 (−2.27, −0.07)	-
HbA1c (%)					
Baseline	8.05 (1.52)	8.10 (1.77)	8.37 (1.44)	8.28 (1.35)	0.463
1-year follow-up	8.47 (1.86)	7.63 (1.89)	7.41 (1.18)	7.27 (1.72)	-
Adjusted changes	0.35 (−0.01, 0.71)	−0.42 (−0.79, −0.06)	−0.90 (−1.27, −0.54)	−1.06 (−1.44, −0.69)	0.000
MD (vs. usual care group)	-	−0.77 (−1.31, −0.23)	−1.25 (−1.79, −0.71)	−1.41 (−1.95, −0.87)	-
MD (vs. diet group)		-	−0.48 (−1.02, 0.06)	−0.64 (−1.19, −0.09)	-
TC (mmol/L)					
Baseline	5.84 (1.83)	4.98 (0.86)	5.04 (0.98)	5.24 (1.03)	0.000
1-year follow-up	6.01 (1.87)	4.8 (1.04)	4.69 (0.95)	4.76 (0.97)	-
Adjusted changes	0.12 (0.07, 0.31)	−0.19 (−0.38, −0.01)	−0.37 (−0.56, −0.18)	−0.49 (−0.68, −0.29)	0.000
MD (vs. usual care group)	-	−0.31 (−0.62, 0.00)	−0.49 (−0.75, −0.23)	−0.61 (−0.88, −0.34)	-
MD (vs. diet group)	-	-	−0.18 (−0.45, 0.09)	−0.30 (−0.57, −0.03)	-
TG (mmol/L)					
Baseline	1.92 (0.94)	1.83 (0.88)	2.06 (1.06)	1.98 (1.00)	0.510
1-year follow-up	2.13 (1.41)	2.00 (1.91)	1.83 (1.19)	1.55 (0.97)	-
Adjusted changes	0.21 (−0.08, 0.50)	0.17 (−0.12, 0.46)	−0.27 (−0.56, 0.02)	−0.45 (−0.75, −0.16)	0.005
MD (vs. usual care group)	-	−0.04 (−0.45, 0.38)	−0.46 (−0.87, −0.05)	−0.66 (−1.07, −0.25)	-
MD (vs. diet group)	-	-	−0.42 (−0.83, −0.01)	−0.70 (−1.11, −0.29)	-
LDL-c (mmol/L)					
Baseline	3.20 (1.05)	2.96 (0.71)	2.90 (0.77)	3.15 (0.85)	0.128
1-year follow-up	3.35 (0.99)	2.84 (0.88)	2.50 (0.79)	2.64 (0.75)	-
Adjusted changes	0.16 (−0.01, 0.33)	−0.14 (−0.30, 0.01)	−0.41 (−0.56, −0.26)	−0.51 (−0.67, −0.35)	0.000
MD (vs. usual care group)	-	−0.30 (−0.51, −0.09)	−0.57 (−0.78, −0.36)	−0.67 (−0.89, −0.45)	-
MD (vs. diet group)	-	-	−0.27 (−0.49, −0.05)	−0.37 (−0.59, −0.15)	-
HDL-c (mmol/L)					
Baseline	1.41 (0.45)	1.30 (0.24)	1.25 (0.21)	1.36 (0.36)	0.022
1-year follow-up	1.39 (0.34)	1.34 (0.57)	1.29 (0.40)	1.39 (0.41)	-
Adjusted changes	−0.02 (−0.11, 0.07)	0.06 (−0.15, 0.03)	0.06 (−0.03, 0.15)	0.01 (−0.09, 0.10)	0.636
MD (vs. usual care group)	-	0.08 (−0.09, 0.26)	0.09 (−0.08, 0.26)	0.03 (−0.15, 0.21)	-
MD (vs. diet group)	-	-	0.00 (−0.17, 0.18)	−0.05 (−0.23, 0.12)	-

* All values were presented as means (SD) or means (95% CI). Changes from the baseline were adjusted for potential confounding variables (sex, age, drinking/smoking, physical activity level, education level, family history, medications and duration of diabetes) in the analysis of covariance model.

## Supplementary Materials

The following is available online at http://www.mdpi.com/2072-6643/8/9/549/s1, Table S1: A sample menus of the general healthy diet provided for intervention groups (average consumption per day).
