# Supplementary material for: Short- and Long-Term Effects of Wholegrain Oat Intake on Weight Management and Glucolipid Metabolism in Overweight Type-2 Diabetics: A Randomized Control Trial
Source: Nutrients. 2016 Sep 7;8(9):549. doi: 10.3390/nu8090549 (PMC5037534; doi:10.3390/nu8090549)
Supplement: Supplementary file 1 [file nutrients-08-00549-s001.docx]

Supplementary Materials: Short- and Long-Term Effects of Wholegrain Oat Intake on Weight Management and Glucolipid Metabolism in Overweight Type-2 Diabetics: A Randomized Control Trial

Xue Li, Xiaxia Cai, Xiaotao Ma, Lulu Jing, Jiaojiao Gu, Lei Bao, Jun Li, Meihong Xu,
Zhaofeng Zhang and Yong Li

**Table S1.** A sample menus of the general healthy diet provided for intervention groups (average consumption per day) **.*

| **Raw Food Material** | **Cooking Method** | **Weight (g)** | | | | |
| --- | --- | --- | --- | --- | --- | --- |
|  |  | **Breakfast** | **Lunch** | **Dinner** | **Extra Food** | **Total** |
| Cereals (rice, wheat, black rice, foxtail millet and oat) and potato | steamed/boiled | 80 | 150 | 120 | 0 | 350 |
| Vegetables and fruits (bitter gourd, cabbage, tomato, green pepper, pumpkin, Cucurbita pepo, carrot, celery, rape, aubergine, lettuce, Chinese chive, spinach, wax gourd, cauliflower, cucumber, Chinese cabbage, bean sprout, cherry tomato, onion and garlic), and fungi | Uncooked/boiled/stir-fried | 100 | 200 | 200 | 100 | 600 |
| Lean meat (pork, beef and chicken) and fish | simmered/stir-fried | 0 | 80 | 80 | 0 | 160 |
| Bean products (Tofu and dried bean curd) | boiled/stir-fried | 15 | 30 | 30 | 0 | 75 |
| Egg | boiled | 60 | 0 | 0 | 0 | 60 |
| Milk | - | 0 | 0 | 0 | 200 | 200 |
| Vegetable oil | - | 0 | 13 | 13 | 0 | 26 |
| Salt | - | 1 | 2.5 | 2.5 | 0 | 6 |

* This table includes the raw food material for a 7-day cyclical dietary menu. The average amount of food consumption is shown for per person per day. A maximum of an extra 10% of daily kcal intake was allowed according to the individual need of participants.
